# A Rab20-Dependent Membrane Trafficking Pathway Controls *M. tuberculosis* Replication by Regulating Phagosome Spaciousness and Integrity

**DOI:** 10.1016/j.chom.2017.04.004

**Published:** 2017-05-10

**Authors:** Laura Schnettger, Angela Rodgers, Urska Repnik, Rachel P. Lai, Gang Pei, Martijn Verdoes, Robert J. Wilkinson, Douglas B. Young, Maximiliano G. Gutierrez

**Affiliations:** 1 Host-Pathogen Interactions In Tuberculosis Laboratory, The Francis Crick Institute, 1 Midland Road, London NW1 1AT, UK; 2 Mycobacterial Systems Biology Laboratory, The Francis Crick Institute, 1 Midland Road, London NW1 1AT, UK; 3 Department of Biosciences, University of Oslo, Blindernveien 31, 0371 Oslo, Norway; 4 Tuberculosis Laboratory, The Francis Crick Institute, 1 Midland Road, London NW1 1AT, UK; 5 Department of Immunology, Max Planck Institute for Infection Biology, Charitéplatz 1, 10117 Berlin, Germany; 6 Radboud Institute for Molecular Life Sciences (RIMLS), Geert Grooteplein 26/28, Nijmegen 6525 GA, the Netherlands

**Keywords:** mycobacterium, tuberculosis, phagosome, macrophage, Rab GTPases, Rab20

## Abstract

The intracellular pathogen *Mycobacterium tuberculosis* (Mtb) lives within phagosomes and also disrupts these organelles to access the cytosol. The host pathways and mechanisms that contribute to maintaining Mtb phagosome integrity have not been investigated. Here, we examined the spatiotemporal dynamics of Mtb-containing phagosomes and identified an interferon-gamma-stimulated and Rab20-dependent membrane trafficking pathway in macrophages that maintains Mtb in spacious proteolytic phagolysosomes. This pathway functions to promote endosomal membrane influx in infected macrophages, and is required to preserve Mtb phagosome integrity and control Mtb replication. Rab20 is specifically and significantly upregulated in the sputum of human patients with active tuberculosis. Altogether, we uncover an immune-regulated cellular pathway of defense that promotes maintenance of Mtb within intact membrane-bound compartments for efficient elimination.

## Highlights

• The spatiotemporal dynamics of the *M. tuberculosis* (Mtb) phagosome were investigated
• A Rab20-dependent pathway regulates endocytic influx into Mtb phagosomes
• Mtb avoids targeting to Rab20-positive phagosomes through its ESX-1 system
• Rab20 is required to maintain Mtb phagosome integrity and for control of Mtb replication

## Graphical Abstract

**Figure:**
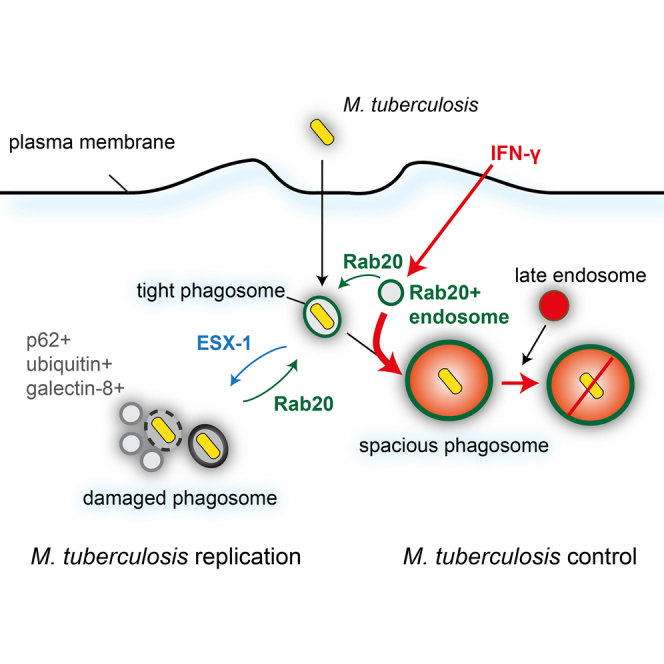

## Teaser

Host-dependent mechanisms that regulate the *M. tuberculosis* (Mtb) phagosome are unexplored. Schnettger et al. investigate the spatiotemporal dynamics of Mtb phagosomes and identify an IFN-γ-stimulated, Rab20-dependent membrane trafficking pathway in macrophages that maintains Mtb in spacious proteolytic phagolysosomes and is required for mycobacterial control.

## Introduction

Mtb has developed multiple strategies to subvert phagosome maturation, thus avoiding the formation of hydrolytic phagolysosomes (Deretic and Fratti, 1999, Vergne et al., 2004a). The proposed molecular mechanisms of phagosome maturation arrest at an early stage have been linked to the retention of the early endocytic marker Rab5, preventing the transition to the more hydrolytic Rab7-positive late phagosomes (Vergne et al., 2004a). However, the use of non-pathogenic strains of various mycobacterial species and the lack of experiments with live cell imaging of *Mycobacterium tuberculosis* (Mtb)-infected cells has precluded comprehensive understanding of the dynamics of Mtb phagosomes in macrophages.

Intracellular pathogens colonizing endomembrane compartments can live in either tight phagosomes where the membrane is mostly attached to bacteria or spacious phagosomes where there is little contact between the bacteria and the limiting membrane (Case et al., 2016). Evidence in tuberculosis (TB) patients and in experimental models shows that Mtb localizes in both tight and spacious phagosomes of host cells (Barisch et al., 2015, Bermudez and Goodman, 1996, Moreira et al., 1997, Rohde et al., 2012, Russell et al., 2002). However, it is not completely understood how these two morphologically different phagosomes are generated and if they are functionally different. Mtb also induces perturbations in the phagosomal membrane via the type VII secretion system ESX-1, which is encoded in the region of difference 1 (RD1). This system allows pathogenic mycobacteria to eventually access the cytosol (Houben et al., 2012, Simeone et al., 2012, van der Wel et al., 2007). Thus, Mtb localizes in different intracellular locations, namely in tight or spacious intact phagosomes, in phagosomes with perturbations in their membranes, or in the cytosol. However, the host cellular machinery that regulates the different populations of intracellular Mtb remains poorly characterized.

Host cells, and in particular mononuclear phagocytes, possess a broad range of anti-microbial pathways to eliminate invading microbes. In macrophages, interferon gamma (IFN-γ) is a cytokine that activates the endocytic, phagocytic, and autophagic pathways (MacMicking, 2014). IFN-γ is known to control Mtb replication by both enhancing phagosome maturation as well as activating autophagy to sequester Mtb into autophagosomes (Gutierrez et al., 2004, Levine and Deretic, 2007). However, Mtb can eventually evade (Kimmey et al., 2015, Watson et al., 2012) as well as induce autophagy under certain conditions (Espert et al., 2015, Mostowy, 2013). Thus, despite the fact that autophagy is clearly important for bacterial control, other as yet uncharacterized IFN-γ-dependent vesicular transport-mediated mechanisms of intracellular Mtb control have been predicted (MacMicking, 2014). In this context, there is compelling evidence showing links between Rab protein expression and intracellular transport after immune activation by IFN-γ (Pei et al., 2012, Pei et al., 2015).

Here, we provide a description of the spatiotemporal dynamics of Mtb phagosome maturation in macrophages. Our study uncovers an IFN-γ-stimulated and Rab20-dependent vesicular trafficking pathway that regulates endosomal interactions with Mtb phagosomes. These interactions reduce phagosomal damage, leading to the retention of Mtb in intact spacious phagosomes that restrict Mtb replication. Thus, our studies reveal a host-dependent immune mechanism of Mtb phagosomal membrane integrity maintenance leading to pathogen control.

## Results

### Spatiotemporal Analysis of Mtb Phagosome Dynamics

**Figure 1:**
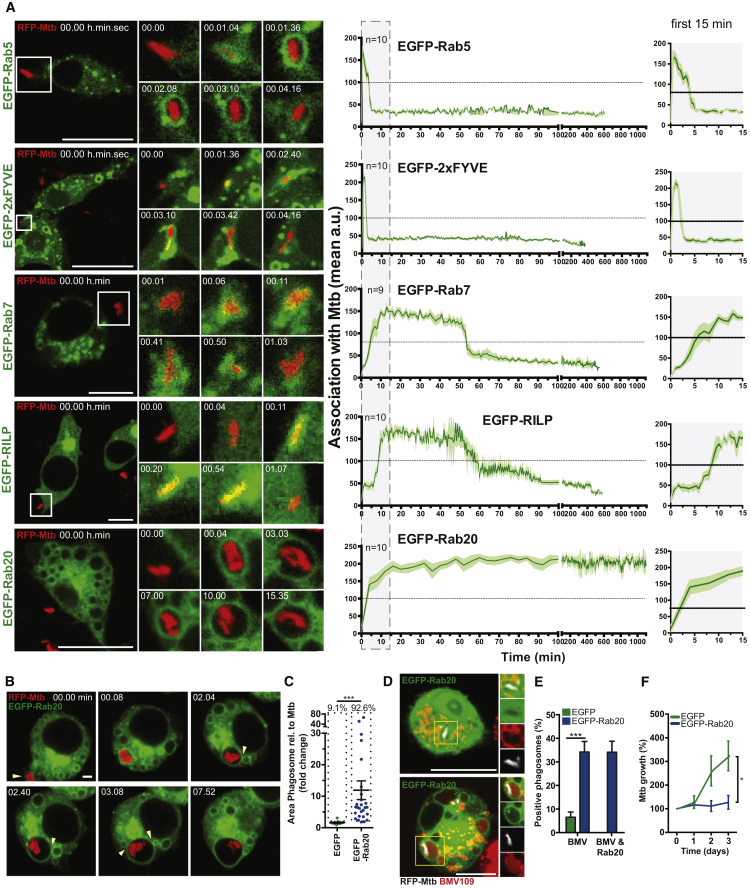
Spatiotemporal Analysis of *M. tuberculosis* Phagosomes and the Role of Rab20 in Spacious Phagosome Generation (A) RAW264.7 macrophages transfected with indicated expression vectors were infected with RFP-Mtb. Images were taken every 32 s or 4 min (EGFP-Rab20) for 24 hr. Quantification for the first 15 min highlighted by the gray box is shown enlarged on the right side of the figure. (B) EGFP-Rab20-expressing cells were infected with RFP-Mtb. White arrowheads indicate fusion of EGFP-Rab20-positive vesicles with the EGFP-Rab20-positive phagosome. (C) Phagosomal area relative to bacterial area in EGFP/EGFP-Rab20 expressing cells at 24 hr post infection. Symbols represent individual phagosomes and the percentages refer to the proportion of spacious phagosomes (i.e., the population within each dotted box). (D) Macrophages infected with RFP-Mtb for 24 hr were incubated with 1 μM BMV109 for 15 min before imaging of live cells. (E) Percentage of BMV109 and BMV109/EGFP-Rab20 double-positive phagosomes in EGFP/EGFP-Rab20-expressing RAW264.7 from (D). (F) RFP-Mtb growth in EGFP-Rab20/EGFP-expressing cells by CFU/mL. Scale bars, 5 μm in all panels. Data are shown as mean relative fluorescent intensity ± SEM from three independent experiments or indicated number of bacteria. Statistical significance was determined using Student's t test (^∗^p < 0.05, ^∗∗∗^p < 0.001). See also Figures S1 and S2; Movies S1 and S2.

To define the dynamics of Mtb phagosome maturation we performed long-term (up to 24 hr) live cell imaging of mouse macrophages infected with RFP-Mtb H37Rv (RFP-Mtb). EGFP-Rab5 associated with RFP-Mtb phagosomes during the first 5 min after internalization (Figures 1A, S1A, and S1E; Movie S1). Endogenous Rab5 was not associated with mycobacterial phagosomes at later time points, either in resting or activated macrophages (Figures S1A–S1C). Consistent with these observations, PtdIns(3)P association, as measured by EGFP-2xFYVE, was transient and occurred only during the first 4 min after phagosome formation (Figure 1A; Movie S1). Conversely, both EGFP-Rab7 and EGFP-RILP associated with RFP-Mtb phagosomes from 6 min and remained associated for approximately 60 min. Overexpressed EGFP-Rab20, which has been shown to associate with early latex bead phagosomes (Pei et al., 2014), associated with RFP-Mtb phagosomes very early but unexpectedly remained associated for 24 hr after infection (Figures 1A, S1E, and S1F; Movie S2). Strikingly, EGFP-Rab20-positive RFP-Mtb phagosomes were significantly enlarged (Figures 1B and 1C; Movie S2), a situation that was not observed with the overexpression of the endocytic Rab GTPases EGFP-Rab5 and EGFP-Rab7 (Figure 1A). These enlarged RFP-Mtb phagosomes were the result of multiple fusion events with EGFP-Rab20-positive endosomes (Pei et al., 2015) (Figure 1B; Movie S2). EGFP-Rab20 expression induced a significant enlargement of RFP-Mtb phagosomes and targeted Mtb to spacious EGFP-Rab20-positive proteolytic phagosomes, as measured by the late endocytic marker LAMP-2 and the activity-based cathepsin probe BMV109 that shows cathepsin-dependent proteolytic activity (Figures 1C–1E, S2A, and S2B) (Verdoes et al., 2013). The formation of Mtb spacious phagolysosomes in EGFP-Rab-expressing macrophages correlated with the restriction of mycobacterial replication (Figures 1F, S2C, and S2D). These results suggest that EGFP-Rab20 promotes membrane influx into tight phagosomes, altering the membrane balance and shifting them into spacious phagosomes that will subsequently fuse with late endocytic compartments.

### Mtb Avoids Targeting to Rab20-Positive Spacious Phagosomes through the ESX-1 System

**Figure 2:**
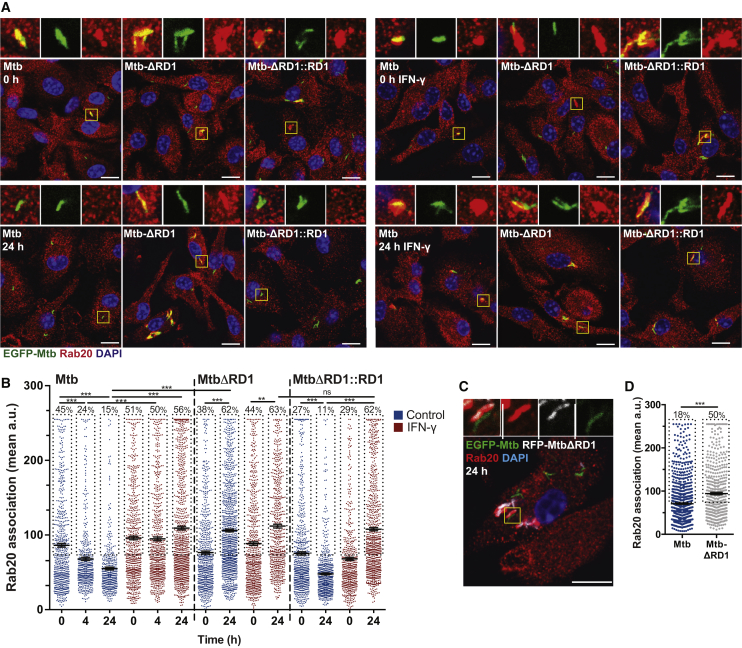
The Interplay between ESX-1 and IFN-γ in the Association of Rab20 with Mtb (A) BMM were incubated with IFN-γ (5 ng/mL) for 24 hr prior to infections. Cells were infected with EGFP-Mtb, EGFP-MtbΔRD1, or EGFP-MtbΔRD1::RD1. Samples were fixed and immunostained for Rab20 and the nuclear stain DAPI. Scale bars, 10 μm. (B) Quantification of Rab20 association with Mtb is shown as mean relative fluorescent intensity ± SEM from three independent experiments. Symbols represent single bacteria or distinct Mtb groups. The percentages refer to the proportion of Rab20+ phagosomes (i.e., the population within each dotted box). (C) BMM were co-infected with EGFP-Mtb and RFP-MtbΔRD1 for 24 hr before fixation and staining for Rab20 and the nuclear stain DAPI. Scale bar, 10 μm. (D) Quantification of Rab20 association with EGFP-Mtb and RFP-MtbΔRD1 at 24 hr in BMM from (C) is shown as mean relative fluorescent intensity ± SEM from three independent experiments. Statistical significance was determined using one-way ANOVA with Dunn's multiple comparison test or Student's t test where appropriate (^∗∗∗^p < 0.001). See also Figure S2.

Given that Rab20 is an IFN-γ-inducible GTPase and that EGFP-Rab20 overexpression in uninfected macrophages mimics the IFN-γ-dependent induction of Rab20 (Pei et al., 2015), we investigated whether IFN-γ is involved in the formation of spacious Mtb phagosomes. In resting macrophages, endogenous Rab20 was present mostly on early Mtb phagosomes and IFN-γ enhanced the association of Rab20 with Mtb phagosomes, especially at 24 hr after infection (Figures 2A and 2B). We then tested if Mtb was able to subvert the IFN-γ-stimulated Rab20 pathway by using a mutant lacking RD1 (EGFP-MtbΔRD1). This mutant is defective in the type VII secretion system ESX-1, which is known to induce phagosomal membrane perturbations (Watson et al., 2012). In resting macrophages, 15% of EGFP-Mtb was in Rab20-positive phagosomes at 24 hr, whereas 62% of the mutants defective in the secretion system ESX-1 (EGFP-MtbΔRD1) were Rab20 positive (Figures 2A and 2B). Confirming a role for ESX-1 in Rab20 dissociation from phagosomes, in macrophages co-infected with EGFP-Mtb and RFP-MtbΔRD1, Rab20 association with RFP-MtbΔRD1 phagosomes was significantly higher within the same co-infected cell (Figures 2C and 2D). Thus, individual phagosomes containing wild-type (WT) bacteria with a functional ESX-1 system were able to subvert the targeting to Rab20-positive phagosomes. This reveals a certain degree of phagosomal autonomy within the same cells (Griffiths, 2004, Hoffmann et al., 2012). Altogether, the data show that in resting macrophages, Mtb is able to evade targeting to Rab20-positive spacious phagosomes through the ESX-1 system.

### Rab20 Is Required for the Generation of Mtb Proteolytic Spacious Phagosomes

**Figure 3:**
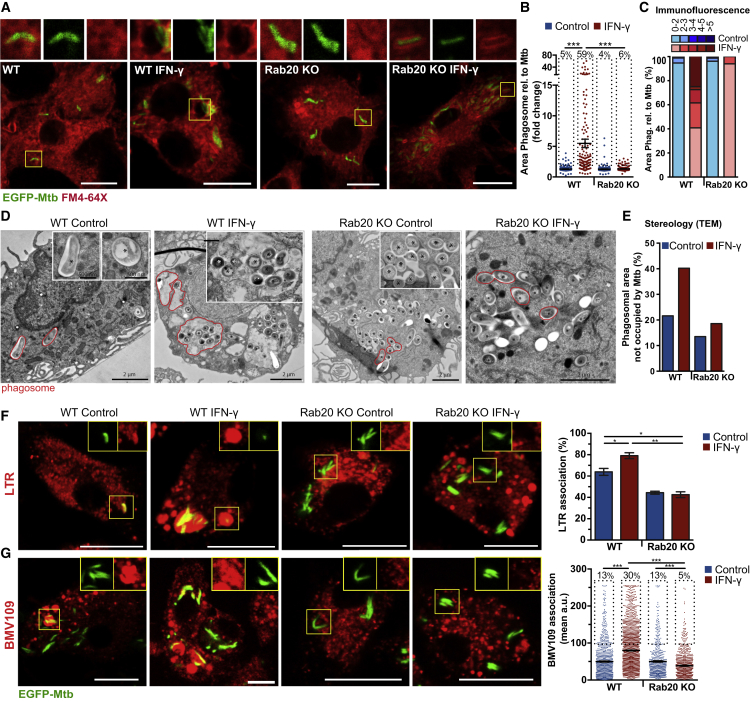
IFN-γ-Dependent Generation of *M. tuberculosis* Spacious Proteolytic Phagosomes Requires Rab20 (A) WT and Rab20 KO BMM infected with EGFP-Mtb for 24 hr were incubated for 5 min with 2.5 μg/mL FM4-64X before fixation. Scale bars, 10 μm. (B) Phagosomal area relative to their bacterial areas from (A). Symbols represent individual phagosomes and the percentages refer to the proportion of spacious phagosomes (i.e., the population within each dotted box). (C) Percentage of spacious phagosomes from (A and B). (D) Representative TEM images of WT and Rab20 KO BMM infected with EGFP-Mtb (^∗^) for 48 hr showing differences in phagosomal size. Examples of tight and spacious phagosomes are outlined in red. (E) Phagosomal volume not occupied by EGFP-Mtb based on stereological analysis from (D). (F and G) BMM infected with EGFP-Mtb for 24 hr were incubated 50 nM LysoTracker-Red (LTR) for 1 hr (F) or 1 μM BMV109 for 15 min (G) before imaging of live cells. Symbols represent single bacteria or distinct Mtb groups. Scale bars, 5 μm. Data are shown as means ± SEM from three independent experiments. The percentages in (G) refer to the proportion of BMV109+ phagosomes (i.e., the population within each dotted box). Statistical significance was determined using one-way ANOVA with Dunn's multiple comparison test (B, G) or Tukey's multiple comparison test (F) (^∗^p < 0.05, ^∗∗^p < 0.01, ^∗∗∗^p < 0.001). See also Figure S3.

The majority of the Rab20-positive spacious phagosomes were also LAMP-2-positive, indicating that spacious Mtb phagosomes had late endocytic features (Figures S2E–S2G). Overall, IFN-γ induced the formation of spacious LAMP-2-positive Mtb phagosomes (Figures S2H–S2J). To understand this better, we compared the area of Mtb phagosomes in bone-marrow-derived macrophages (BMM) from WT and Rab20 knockout (KO) mice (Figures S3A–S3D) by either labeling intracellular membranes with the dye FM4-64FX (Touret et al., 2005) (Figures 3A–3C) or by ultrastructural analysis (Figures 3D and 3E). By measuring the area of each phagosome relative to Mtb, we confirmed that in WT BMM, IFN-γ induced a significant increase in the area of EGFP-Mtb-containing phagosomes. This effect of IFN-γ on Mtb phagosome enlargement was completely abolished in Rab20 KO BMM, indicating that Rab20 was not only a marker for spacious phagosomes but also actively required for their formation. The total number of spacious phagosomes, defined as area ≥2, was also significantly higher after IFN-γ activation (5.4% control versus 59.2% IFN-γ treated) and this effect was absent in Rab20-deficient macrophages (3.8% control versus 6.0% IFN-γ treated) (Figures 3A–3C). Thus, IFN-γ enhanced targeting of Mtb to enlarged phagosomes and this increase in phagosomal size required Rab20. We next characterized the nature of the spacious Mtb phagosomes. In WT BMM, IFN-γ targeted Mtb to LAMP-2-positive phagosomes (Figures S3D and S3E), confirming our previous result (Figures S2E–S2G). This targeting of Mtb to LAMP-2-positive phagosomes by IFN-γ was dependent on Rab20 since macrophages lacking Rab20 show reduced targeting to late phagosomes (Figures S3D and S3E). In agreement with this result, IFN-γ also increased the percentage of LysoTracker-positive Mtb phagosomes in a Rab20-dependent manner (Figure 3F). Importantly, when we used the fluorescently quenched, activity-based probe BMV109 to monitor proteolytic activity, we observed an IFN-γ/Rab20-dependent increase in the association of Mtb phagosomes with protease-positive compartments (Figure 3G). These data show that the IFN-γ-stimulated, Rab20-dependent spacious Mtb phagosomes are acidic, proteolytic, and interact with late endocytic organelles.

### Rab20 Is Required to Maintain Mtb Phagosome Membrane Integrity

**Figure 4:**
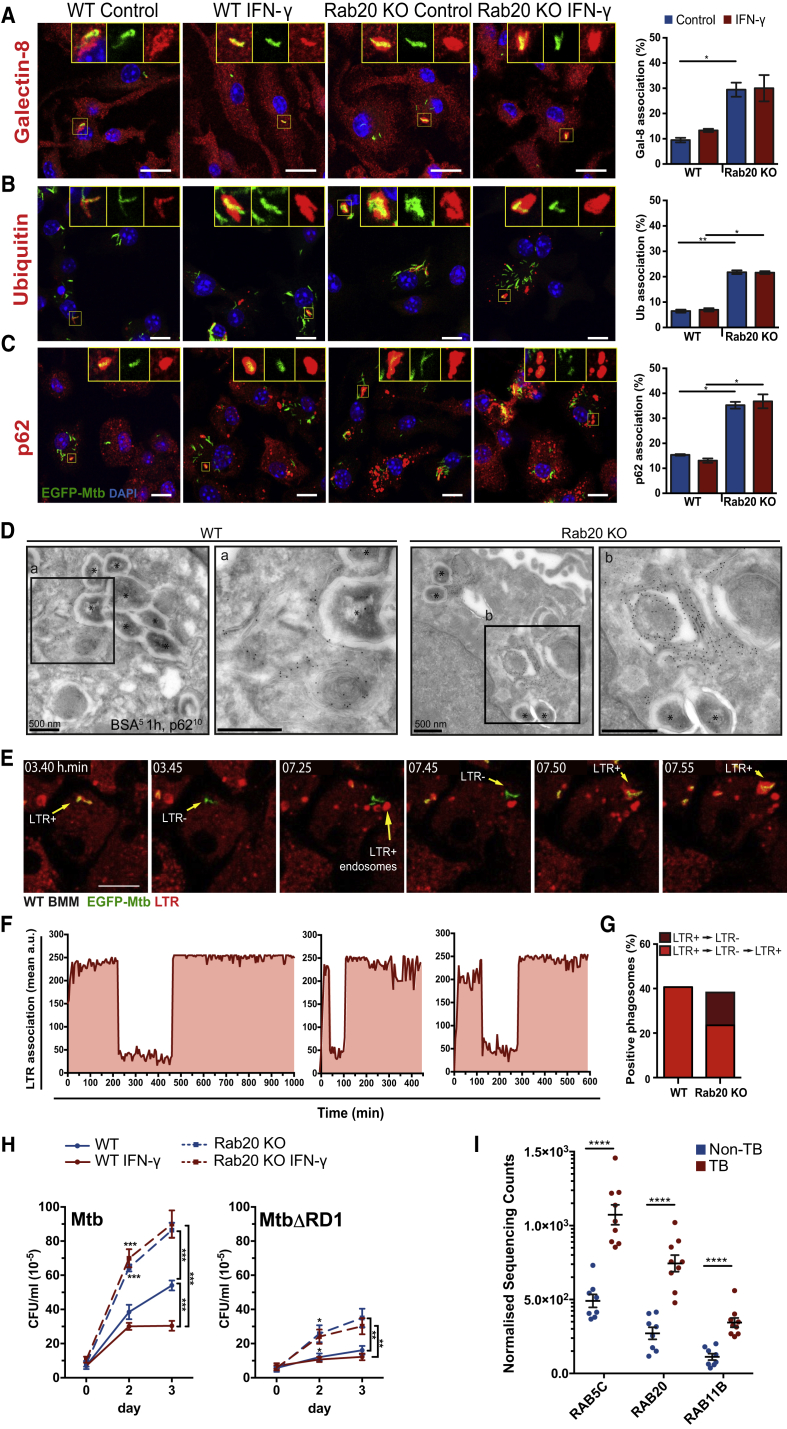
Rab20 Is Required for Maintenance of *M. tuberculosis* Phagosome Integrity and Control of Bacterial Replication (A–C) WT and Rab20 KO BMM were infected with EGFP-Mtb for 24 hr and immunostained for galectin-8 (A), ubiquitin (B), p62 (C) and DAPI. Scale bars, 10 μm. (∗p < 0.05, ∗∗p < 0.01). (D) Representative images of immunogold labeling of p62 on Tokuyasu thawed cryosections of BMM infected with EGFP-Mtb (^∗^) for 48 hr. (E) BMM were incubated with 50 nM LysoTracker-Red (LTR) for 1 hr before infection with EGFP-Mtb. Images were taken every 5 min for 24 hr. Three independent experiments were performed. Scale bars, 5 μm. (F) Quantification of dynamic LTR association with three individual EGFP-Mtb in WT BMM measured in a two-pixel wide ring around the region corresponding to bacteria. (G) Percentage of EGFP-Mtb association with LTR over 24 hr. Quantification is shown from one representative experiment out of three. (H) Growth of EGFP-Mtb/EGFP-MtbΔRD1 in WT and Rab20 KO BMM by CFU/mL. Statistical analysis at day 2 compares resting and IFN-γ-stimulated conditions. Data are shown as means ± SEM from three independent experiments. Statistical significance was determined using one-way ANOVA with Tukey's multiple comparison test or Student's t test where appropriate (^∗^p < 0.05, ^∗∗^p < 0.01, ^∗∗∗^p < 0.001). (I) RNA-seq counts of the top three Rab GTPases with differences in sputum samples from TB and non-TB patients. Symbols represent individual patients; statistical significance was determined using Wald test adjusted for Benjamini-Hochberg multiple testing (^∗∗∗∗^p < 0.00001). See also Figure S4 and Movie S3.

Given that the ESX-1 system promotes phagosomal membrane perturbations and autophagic targeting (Watson et al., 2012), we tested if the ESX-1-dependent evasion of the Rab20 pathway was related to phagosomal membrane perturbations. Consistent with this hypothesis, an increased recognition of Mtb phagosomes by the receptor of vesicle damage, galectin-8, was observed in Rab20 KO relative to WT macrophages (Figure 4A). This recognition by galectin-8 was RD1 dependent (Figure S3F), suggesting that host membranes were damaged via ESX-1, with subsequent exposure of host phagosomal glycans to the cytosol (Thurston et al., 2012). We reasoned that if mycobacteria or mycobacterial components were able to access the cytosol, they might be recognized by molecules of the selective autophagy pathway such as ubiquitin and p62 (SQSTM1) (Watson et al., 2012). In WT BMM there was some localization of ubiquitin (6.5%) and p62 (15%) with Mtb (Figures 4B and 4C) that was entirely dependent on RD1 (data not shown), as previously shown (Watson et al., 2012). Conversely, in Rab20 KO BMM, the percentage of bacteria positive for ubiquitin and p62 was significantly higher (22% and 35%, respectively) (Figures 4B and 4C). The increased association of galectin-8, ubiquitin, and p62 with Mtb was independent of IFN-γ activation (Figures 4A–4C) and likely induced by Mtb-dependent membrane damage. Confirming these observations, the ultrastructure of infected Rab20 KO BMM more often showed multiple p62-labeled host-derived membranes and vesicles in close proximity to Mtb compared with WT BMM (Figure 4D). To monitor phagosome integrity dynamics in macrophages, we used LysoTracker as a probe that, after irreversible protonation, gets trapped into the lumen of acidic compartments (Yan et al., 2016). We observed that Mtb phagosomes became leaky for LysoTracker at different stages in WT BMM and fusion with endosomes restored LysoTracker levels within phagosomes (Figures 4E–4G; Movie S3). EGFP-MtbΔRD1 phagosomes, which were mostly LysoTracker-positive, were not leaky (Figure S3H). Notably, in Rab20 KO BMM, the number of leaky phagosomes that did not retain the probe during 24 hr after infection was significantly higher, with a 2-fold reduction of leaky phagosomes that were able to recover membrane integrity (Figures 4G and S3G). Altogether, Rab20-deficient macrophages have a pronounced defect in Mtb phagosome membrane integrity maintenance.

### Rab20 Is Required for Mtb Control by Macrophages

To understand the functional consequences of Rab20-dependent Mtb retention in intact spacious phagosomes, we used a small hairpin RNA (shRNA)-based strategy (Pei et al., 2014) and found that, in RAW264.7 macrophages knocked down for Rab20, RFP-Mtb replication was significantly higher than in macrophages expressing scrambled shRNA (Figures S4A–S4C). EGFP-Mtb replication in WT BMM during the first 3 days after infection was restricted by IFN-γ treatment (Figures 4H and S4D). Conversely, in Rab20 KO BMM, bacterial replication was uncontrolled and the effect of IFN-γ on bacterial growth restriction was impaired. Although EGFP-MtbΔRD1 growth was minimal in both resting and IFN-γ-activated WT BMM, EGFP-MtbΔRD1 replication was uncontrolled in Rab20 KO BMM (Figures 4H and S4D). EGFP-Mtb burden in vivo was higher in the lungs of Rab20 KO mice only at 7 days after infection (Figure S4E). As expected, EGFP-MtbΔRD1 burden in lungs was lower relative to mice infected with WT EGFP-Mtb. However, the EGFP-MtbΔRD1 burden in lungs was higher in Rab20 KO mice relative to control mice for up to 50 days after infection (Figure S4E). Accordingly, the number of lesions was higher in Rab20 KO mice infected with EGFP-MtbΔRD1 (Figures S4E and S4G). Overall, the in vitro and in vivo data suggest that WT Mtb is able to effectively evade the early targeting to Rab20-positive phagolysosomes. Conversely, MtbΔRD1 is continuously in intact phagosomes and targeted by the Rab20 pathway, leading to continuous growth restriction. Finally, by analyzing sputum from TB and non-TB patients using RNA sequencing, we identified Rab20 as one of the top three Rab GTPases upregulated specifically in patients with active TB (Figure 4I).

## Discussion

Different from previous studies (Fratti et al., 2001, Vergne et al., 2004b, Via et al., 1997), we found that in mouse macrophages, Mtb phagosomes initially follow a conventional maturation pathway, including an early EGFP-Rab5 to EGFP-Rab7 transition followed by dissociation. The transient association of EGFP-Rab7 suggests that Mtb faces temporally late endosomal stages during the first hour after infection. A similar transient association of Rab7, but not RILP, was observed during infection of macrophages with BCG and Mtb in fixed cells (Seto et al., 2011, Sun et al., 2007). Clearly, because of the slow replication time of Mtb, events occurring after the first hour of infection will be of importance for either Mtb growth or restriction. On the other hand, Rab20 remains associated with Mtb phagosomes and mediates membrane influx altering the membrane and generating spacious phagosomes that will fuse with late endocytic compartments. The occurrence of tight and spacious Mtb phagosomes are conserved across host species and likely to be relevant in vivo since these spacious phagosomes have been observed in monocytes of TB patients (Russell et al., 2002), as well as in infected mice (Moreira et al., 1997), zebrafish (Hosseini et al., 2014), and *Dyctiostelium* (Barisch et al., 2015).

Our results are consistent with a mechanism in which disruption of the close contact between the phagosomal membrane and the bacteria will result in enhanced fusion with late endocytic organelles (de Chastellier et al., 2009, Rhoades and Ullrich, 2000). We propose that tight phagosomes preferentially allow the type VII secretion system to be active and facilitate the access of Mtb to the cytosol. Thus, endocytic membrane influx into Mtb phagosomes could be an effective physical mechanism to reduce bacterial secretion via the type VII secretion system, in agreement with original reports in alveolar macrophages showing membrane disruption at the close contacts of the phagosomal membrane with bacilli (Myrvik et al., 1984). IFN-γ, by enhancing interactions with early endocytic vesicles in a Rab20-dependent manner, restricted Mtb growth arguing that host cells are able to maintain Mtb “phagosomally trapped” in a spacious membranous compartment (MacMicking, 2014). Although the presence of galectin-8 implies that at least a part of the phagosome and/or mycobacteria must be exposed to the cytoplasm, we were unable to identify cytosolic bacteria by conventional electron microscopy during the first 2 days of infections, as reported previously (Behar et al., 2010) (and data not shown). At the early stages of infection, Mtb has not been observed to localize in the cytosol in mouse macrophages, but time-dependent phagosomal membrane damage via ESX-1 and release of bacterial DNA has been reported (Watson et al., 2012). Our data argue that in mouse macrophages phagosomal membrane damage can occur. By increasing the amount of membrane influx into phagosomes, Rab20 contributes to maintaining Mtb in a membrane-bound compartment and reducing the number of phagosomes with cytosolic access (Thurston et al., 2012, Watson et al., 2012). We propose that Mtb in tight phagosomes alters host membranes resulting in local ubiquitination. Either host membranes or mycobacterial components will be ubiquitinated and recognized by the selective autophagy machinery. However, the contribution of pathogen-induced ubiquitin and p62 networks of membranes to bacterial replication remains to be defined. Our data suggest that, in cells lacking Rab20, selective autophagy can be beneficial for Mtb replication. Probacterial autophagy has been reported for different bacteria including Mtb (Andersson et al., 2016, Lerena and Colombo, 2011, Lerner et al., 2016, Mostowy, 2013). It is also possible that while autophagic proteins are recruited to Mtb, autophagosome fusion with lysosomes is impaired by this pathogen, as shown previously (Espert et al., 2015, Romagnoli et al., 2012). Rab20 maintains Mtb phagosomal membrane integrity and hence likely only indirectly plays a role in the recognition of phagosomal membrane damage by selective autophagy. The in vivo data suggest that WT Mtb is able to effectively evade the Rab20-dependent targeting to phagolysosomes early, whereas MtbΔRD1 is continuously in intact phagosomes and targeted by the Rab20 pathway, leading to growth restriction. Moreover, the early in vivo differences argue that Rab20 contributes to the innate immune response to Mtb. Our data are in agreement with the requirement of innate antigen-independent IFN-γ secretion for early protective immunity against Mtb (Feng et al., 2006, Kupz et al., 2016). The modest differences in bacterial burdens in lungs of Rab20 KO mice are likely due to TB susceptibility being controlled by more than one single trait (Flynn, 2006) and important pathways such as nitric oxide production still being functional in Rab20 KO mice. Clearly, Rab20 is part of the host response during human TB because it is specifically upregulated in patients with active disease. This finding, together with Rab20 being the only Rab GTPase present in the blood transcriptional signature of TB (Berry et al., 2010), argues that Rab20 is likely an important player in human TB. Altogether, our findings identify an intracellular pathway of phagosome integrity maintenance that could be important to control bacterial pathogens that access the cytosol of host cells.

## STAR★Methods

### Key Resources Table

**Table:** 

REAGENT or RESOURCE	SOURCE	IDENTIFIER
**Antibodies**		

Rabbit polyclonal anti-Rab20 (N1C3)	GeneTex	Cat# GTX119559; RRID: AB_10617706
Rabbit polyclonal anti-Rab5	Cell Signaling	Cat# 3547; RRID: AB_10828212
Mouse polyclonal anti-Rab5	BD Biosciences	Cat# 610282; RRID: AB_397677
Mouse monoclonal anti-EEA1	BD Biosciences	Cat# 610457; RRID: AB_397830
Rabbit monoclonal anti-Syntaxin6	Cell Signaling	Cat# 2869; RRID: AB_10829116
Rat monoclonal anti-LAMP-2	Developmental Hybridoma bank	Cat#abl-93; RRID: AB_2134767
Goat polyclonal anti-Galectin-8	R&D Systems	Cat#AF1305; RRID: AB_2137229
Mouse monoclonal anti-Ubiquitin	Enzo	Cat# BML-PW8810; RRID: AB_10541840
Rabbit polyclonal anti-SQSTM1(p62)	GeneTex	Cat# GTX111393; RRID: AB_10723101

**Bacterial and Virus Strains**		

EGFP-Mtb H37Rv	Lerner et al., 2016	N/A
EGFP-MtbΔRD1 H37Rv	Lerner et al., 2016	N/A
EGFP- MtbΔRD1::RD1 H37Rv	Lerner et al., 2016	N/A
RFP-Mtb H37Rv	Lerner et al., 2016	N/A
*M. bovis* BCG str. Pasteur 1173P2 expressing GFP (GFP-BCG)	Laboratory of Douglas Young	N/A

**Chemicals, Peptides, and Recombinant Proteins**		

Recombinant mouse Interferon-gamma	R&D Systems	Cat# 485-MI-100
Geneticin (G418)	Life technologies	Cat# 10131027
Puromycin	Sigma-Aldrich	Cat# P8833
BMV109	Verdoes et al., 2013	N/A
LysoTracker-DND99	Life technologies	Cat# L7528
FM4-64X	Life technologies	Cat# F34653
Middlebrook’s 7H9 broth medium	Sigma-Aldrich	Cat# M0178
Middlebrook’s 7H11 agar medium	Sigma-Aldrich	Cat# M0428
Middlebrook OADC	BD Biosciences	Cat# 212351
Middlebrook ADC	BD Biosciences	Cat# 212352

**Experimental Models: Cell Lines**		

Mouse macrophage cell line RAW264.7	ATCC	Cat# TIB-71

**Experimental Models: Organisms/Strains**		

Mouse: C57BL/6J (wild-type)	Bred in house	N/A
Mouse: C57BL/6J^tm1a (EUCOMM)Hmgu^ mice (Rab20 KO)	This paper	C57BL/6J^tm1a (EUCOMM)Hmgu^

**Oligonucleotides**		

Primer set1 for mouse genotyping: *Rab20-5*′-*arm:* AGCTCCTTGCGCTCCTCCTCATGG *Exon2:* CAGATGTGCTTCGAGACCAGTGCC	This paper	N/A
Primer set2 for mouse genotyping: *Rab20-5*′*arm:* AGCTCCTTGCGCTCCTCCTCATGG *Insert:* CAACGGGTTCTTCTGTTAGTCC	This paper	N/A
Primer set3 for mouse genotyping: *Exon2:* CAGATGTGCTTCGAGACCAGTGCC *Rab20-3*′-*arm:* GCAGACTCTGAGGAAATGAGATGG	This paper	N/A

**Recombinant DNA**		

Plasmid: pEGFP-C1	Clontech	
Plasmid: pEGFP-Rab20	Pei et al., 2014	N/A
Plasmid:p EGFP-Rab20T19N	Pei et al., 2014	N/A
Plasmid: pEGFP-Rab5A	Pei et al., 2014	N/A
Plasmid: pEGFP-2xFYVE	Pei et al., 2014	N/A
Plasmid: pEGFP-Rab7	Pei et al., 2014	N/A
Plasmid: pEGFP-RILP	(Terebiznik et al., 2006)	N/A
Plasmid: MISSION pLKO.1-puro-Control	Pei et al., 2014	N/A
Plasmid: MISSION pLKO.1-puro-shRNA2643	Pei et al., 2014	N/A
Plasmid: MISSION pLKO.1-puro-shRNA2644	Pei et al., 2014	N/A
Mycobacterial plasmid: pML1335 (EGFP)	Lerner et al., 2016	N/A
Mycobacterial plasmid: pML2570 (RFP)	Lerner et al., 2016	N/A

**Software and Algorithms**		

Prism	GraphPad Software	https://www.graphpad.com/scientific-software/prism/
ImageJ/Fiji	NIH, Bethesda/US	https://fiji.sc/

**Deposited Data**		

Sequencing data (PRJEB10919)	European Nucleotide Archive	http://www.ebi.ac.uk/ena

### Contact for Reagent and Resource Sharing

Further information and requests for resources and reagents should be directed to and will be fulfilled by the Lead Contact, Maximiliano G. Gutierrez, (Max.G@crick.ac.uk)

### Experimental Model and Subject Details

#### Ethics Statement

All animals were bred and maintained for experiments in accordance with the United Kingdom Home Office regulations. All experimental protocols were approved by the United Kingdom Home Office (project license 70/8045). Clinical samples collection from human patients was approved by the Human Research Ethics Committee of the University of Cape Town (HREC References: 031/2012 and 568/2012) and written informed consent was obtained from all participants.

#### Mouse Experiments

C57BL/6J (WT) and C57BL/6J^tm1a (EUCOMM)Hmgu^ mice (Rab20 KO) were bred and housed under specific pathogen-free conditions at the Francis Crick Institute. Groups (n=5) of female 6-8 week old mice were used for experiments.

#### Cell Culture Experiments

Mouse bone marrow macrophages (BMM) were isolated from 6-8 week old C57BL/6J (WT) and C57BL/6J^tm1a (EUCOMM)Hmgu^ mice (Rab20 KO). BMM and RAW264.7 macrophages (ATCC, #TIB-71) were cultured in 37 °C, 5% CO_2_ atmosphere.

#### Human TB patients

Spontaneous sputum samples were collected from a total of 17 patients, 9 of which were confirmed to have active TB disease by smear AFB staining and/or gene Xpert. The 8 non-TB patients were respiratory symptomatic but were excluded of TB as confirmed by smear AFB staining, gene Xpert, sputum culture and/or radiography. None of the TB patients was on antitubercular therapy at the time of sample collection. Patients in both TB and non-TB groups were matched in age and gender.

### Method Details

#### Isolation of Mouse Bone Marrow Macrophages

Primary mouse bone marrow macrophages were isolated as described (Schnettger and Gutierrez, 2017). The bone marrow of mice from the C57BL/6J background was isolated and the cells were plated on sterile microbiology uncoated 9 cm petri dish in RPMI supplemented with 10 % FCS and 20 % L929 fibroblast supernatant. Cells were differentiated for 6 days at 37 °C in 5% CO_2_ atmosphere with replacement of 70-80 % of media every 48 h. BMM were collected in ice-cold PBS and plated in RPMI + 10 % FCS for experiments.

#### RAW264.7 Macrophage Cell Culture

The mouse macrophage cell line RAW264.7 (ATCC, #TIB-71) was maintained in high glucose, L-glutamine containing Dulbecco’s Modified Eagles Medium (DMEM) supplemented with 10 % heat inactivated fetal calf serum (FCS, Gibco) without addition of antibiotics. For CFU assays EGFP or EGFP-Rab20WT expressing RAW264.7 were cultured with the addition of geneticin (30 μg/ml, Life technologies) and RAW264.7 cells expressing shRNA against Rab20 (2644/2643) were cultured with the addition of puromycin (Sigma). Mouse bone marrow derived macrophages (BMM) were obtained as previously described (Bronietzki et al., 2014, Schnettger and Gutierrez, 2017). Cells were incubated at 37 °C, 5 % CO_2_. For the experiments, macrophages were prestimulated 24 h before infection with IFN-γ (5 ng/ml, R&D Systems).

#### Bacterial Strains and Culture

The different strains of H37Rv *M. tuberculosis* used in this study were previously described (Lerner et al., 2016). All strains were genome sequenced to confirm their identity prior to use. *M. bovis* BCG str. Pasteur 1173P2 expressing GFP (GFP-BCG) was kindly provided by Dr Douglas Young (The Francis Crick Institute, UK). Mycobacteria were cultured in Middlebrook’s 7H9 broth medium (Sigma-Aldrich, M0178) supplemented with 10 % (v/v) Middlebrook OADC (BD Biosciences, 212351) and 0.05 % (v/v) Tween80 (Sigma-Aldrich, P1754) and incubated at 37°C with rotation in 50 ml Falcon tubes (cultures up to 10 ml). Mycobacteria were also cultured on Middlebrook’s 7H11 agar medium (Sigma-Aldrich, M0428) supplemented with 10 % (v/v) Middlebrook OADC and incubated at 37 °C for 2 to 3 weeks until colonies appeared.

#### Macrophage Transfection

RAW264.7 macrophages were transfected with jetPEI DNA transfection reagents (Polyplus-transfection). 2x10^5^ cells were plated on 10 mm glass coverslips (Assistent) in 24-well plates 6 h before transfection with 1 μg DNA and 2 μl jetPEI. Cells were transfected 16-20 h before further experiments.

#### Infection of Macrophages with M. tuberculosis

For macrophage infections mycobacteria were prepared as previously described (Lerner et al., 2016) (Schnettger and Gutierrez, 2017). Mtb cultures were grown to midexponential phase (OD_600_ =0.6±0.2). Bacterial clumps were separated by vigorously shaking with an equivalent volume of sterile 2.5-3.5 mm glass beads. Bacteria were resuspended in cell culture media and the remaining clumps were removed by centrifugation at 300 g for 5 min. Macrophages were infected with Mtb at a multiplicity of infection (MOI) of 10 (RAW264.7) or 2 (BMM) for 2 h. These MOIs added to RAW264.7 and BMM hereby resulted in the internalisation of a similar number of bacteria (1-2) per cell. No differences in the internalisation of Mtb by WT and Rab20 KO BMM were observed by CFU, fluorescent signal per cell and percentage of infected cells. Cells were washed twice with PBS and chased for indicated time in complete DMEM (RAW264.7) or RPMI (BMM). For experiments analysing Mtb growth in BMM by CFU and EFGP signal/cell macrophages were infected with an MOI of 0.5. A minimum of 3 independent experiments was performed with BMM isolated from different animals and Mtb grown from stocks to mid-exponential phase separately for each experiment.

#### Live Cell Imaging

For analysis of the dynamic association of phagosomal markers to Mtb, RAW264.7 macrophages were plated at 2x10^5^ on WillCo-dish glass-bottom dishes (WillCo Well BV) and transfected one day prior to infection (Schnettger and Gutierrez, 2017). Cells were infected with Mtb at a MOI of 2. Imaging was performed using a Leica TCS SP5 II microscope (Leica Microsystems) equipped with AOBS, a HC PLAOP CS2 63.0x1.40 OIL objective and an environmental control chamber providing 37°C, 5 % CO_2_ and 20-30 % humidity in a closed chamber during imaging. Images were acquired in 32 sec or 4 min intervals over a time frame of 24 h. For analysis of association of different probes with Mtb phagosomes at 24 h in live cells, RAW264.7 or BMM were incubated with 1 μM BMV109 for 15 min, 2.5 μg/ml FM4-64X (Life Technologies) for 5 min or 50 nM LysoTracker-DND99 (Life Technologies) for 1 h prior to imaging of live cells. A minimum of 3 independent experiments was performed.

#### Indirect Immunofluorescence

Macrophages were plated on 10 mm glass coverslips (Assistent). Samples were washed with PBS prior to fixation with 3 % paraformaldehyde (PFA, Electron Microscopy Sciences) in PBS overnight at 4 °C. Cells were washed twice in PBS and subsequently quenched in 50 mM NH_4_Cl/PBS for 10 min, followed by permeabilization with 0.05 % Saponin (Sigma), 1 % bovine serum albumin (BSA, Sigma-Aldrich) in PBS for 10 min. Samples were incubated in 1 % BSA/PBS for 5 min prior to incubation with primary and secondary antibodies in 1 % BSA/PBS for 1 h. Three washing steps with PBS for 5 min followed each antibody incubation. The nuclear stain DAPI (Life technologies, 300 nM) was applied for 10 min at room temperature. Glass coverslips were mounted on glass slides (Thermo Scientific) using Fluorescent mounting medium (Dako Cytomation). The following primary and secondary antibodies were used: Rab20 (N1C3, GeneTex), LAMP-2 (abl-93, Developmental Studies Hybridoma bank), Rab5 (3547, Cell Signaling), p62 (SQSTM1, GeneTex), ubiquitin (FK2, Enzo), Galectin-8 (R&D Systems), EEA1 (BD Biosciences), Syntaxin6 (Cell Signaling), rabbit-Cy3 (Alpha Diagnostics International), rabbit-AlexaFluor 488 (Invitrogen), mouse-AlexaFluor 488 (Invitrogen), rat-AlexaFluor 546 (Invitrogen), goat-Cy3 (Alpha Diagnostics International) and rat-Cy5 (Alpha Diagnostics International). Images of the samples were acquired with blinding of the experimental conditions. A minimum of 3 independent experiments with at least 200 phagosomes per condition in each experiment was analysed.

#### Western Blot Analysis

Samples were collected and the cell pellet was lysed in cold Nonidet-P40 (NP40) lysis buffer (150 mM NaCl, 50 mM Tris pH8.0, 1 % NP-40, protease inhibitor cocktail (Roche)) on ice for 40 min. The samples were boiled at 95 °C for 10 min together with Sample Buffer and reducing agent (NuPAGE, Life Technologies) and run on a NuPAGE 4-12% Bis-Tris gel (Life Technologies) together with a pre-stained protein ladder (BLUeye Prestained Protein Ladder, Geneflow). The gel was transferred onto a nitrocellulose membrane using the iBlot2 Blotting System (Life Technologies, 7 min). The membranes were blocked in 6 % semi-skinned milk (Invitrogen) in PBS-T (PBS, 0.1 % Tween-20 (MP Biomedicals LLC)). The membranes were incubated with primary and secondary antibodies in 6 % skimmed milk in PBS-T at 4 °C overnight and 1 h at room temperature, respectively. Antibodies used: Rabbit anti-Rab20 (Proteintech), Rabbit anti-GAPDH (Sigma) and Goat anti-rabbit-HRP (Promega). A minimum of 3 independent experiments with each two technical replicates was analysed.

#### Ultrastructural Analysis

WT and Rab20KO BMM were plated at 1.5x10^6^ cells in T25 flasks. BMM were fed with 5 nm gold (OD_600_ 3) for 1 h, washed twice with 1 % BSA/PBS followed by incubation in complete medium for 1 h. Macrophages were then infected with EGFP-Mtb/ EGFP-MtbΔRD1 at a MOI of 0.5 for 2+48 h. For sample fixation warm 2 % glutaraldehyde (EMS) or 8 % PFA (EMS) in 400 mM HEPES, pH7.4, was added directly to the cell culture medium at a 1:1 volume ratio for 5 min. The fixative medium mixture was replaced by 1 % glutaraldehyde or 4 % PFA in 200 mM HEPES buffer for fixation of cells for 30 min at room temperature followed by overnight incubation at 4°C. Glutaraldehyde fixed cells were embedded in Spurr’s resin and sections prepared as described previously (Lerner et al., 2016). For the stereological analysis a minimum of 24 profiles of different infected cells was analysed per sample by counting cross-points of the stereological test grid over bacteria and over the phagosomal lumen. The relative volume density of bacteria in phagosomes was calculated as a fraction of the total count of cross-points over bacteria relative to the total count of cross-points over the phagosomal lumen. PFA fixed cells were prepared for Tokuyasu sectioning and immunogold labeling as described previously (Lerner et al., 2016). Tokuyasu sections were incubated with mouse anti-p62 (Abnova) at 1:100 for 20 min, followed by rabbit anti-mouse (DAKO) at 1:200 for 20 min and PAG10 (The Cell Microscopy Center, University Medical Center, Utrecht) at 1:50 for 30 min. Both resin and Tokuyasu sections were examined with JEM1400 transmission electron microscope (JEOL). Images were taken with TemCam-F216 (Tvips).

#### CFU Assay

For CFU assays RAW264.7 cells expressing shRNA against Rab20 (2644/2643) or WT and Rab20 KO BMM were infected at a MOI of 10 or 0.5 respectively. After the indicated time points, macrophages were washed once in PBS and intracellular bacteria were released by cell lysis in sterile water containing 0.05 % Tween80 for 1 h at room temperature. Samples from triplicate wells were serially diluted in PBS and plated on triplicate 7H11 agar plates. Agar plates were incubated at 37°C for 2-3 weeks and colonies were counted. CFU were plotted as the average CFU per ml from 3 biological replicates with each 3 technical replicates.

#### Mouse Infections

C57BL/6J (WT) and C57BL/6J^tm1a (EUCOMM)Hmgu^ mice (Rab20 KO) were bred and housed under specific pathogen-free conditions at the Francis Crick Institute. All animals were bred and maintained for experiments in accordance with the United Kingdom Home Office regulations. All experimental protocols were approved by the United Kingdom Home Office (project license 70/8045). Groups of female 6-8 week old mice were infected by low-dose aerosol exposure with a growing (mid log phase) culture of EGFP-Mtb H37Rv and EGFP-MtbΔRD1 H37Rv (Lerner et al., 2016) using a Glas-Col (Terre Haute, IN) aerosol generator calibrated to deliver approximately 100 bacteria into the lungs. Bacterial counts in the lungs of 5 animals at each time point of the study were determined by plating serial dilutions of individual organ homogenates on duplicate plates of Middlebrook 7H10 agar containing OADC enrichment. CFU were counted after 3–4 weeks incubation at 37 °C.

#### Immunopathology of Mtb Infected Mice

Lungs were harvested from C57BL/6J (WT) and C57BL/6J^tm1a (EUCOMM)Hmgu^ (Rab20 KO) mice infected with EGFP-Mtb and EGFP-MtbΔRD1 for 30 days. Lungs were perfused in 4 % PFA for a minimum of 24 h followed by transfer to 70 % ethanol. Lungs were paraffin embedded, section and stained for haematoxylin and eosin (H&E). Lung sections were scanned with an Olympus Virtual Slide Microscope VS120 equipped with a 40x objective. Lung inflammation in EGFP-Mtb infected mice was analysed by measuring inflamed dense area in two lung sections per mouse using the image analysis software ImageJ. In EGFP-MtbΔRD1 infected mice the number of lesions per lung section was counted.

#### Sputum Collection and RNA Sequencing

Spontaneous sputum samples were collected from a total of 17 patients, 9 of which were confirmed to have active TB disease by smear AFB staining and/or gene Xpert. The 8 non-TB patients were respiratory symptomatic but were excluded of TB as confirmed by smear AFB staining, gene Xpert, sputum culture and/or radiography. None of the TB patients was on antitubercular therapy at the time of sample collection. Patients in both TB and non-TB groups were matched in age and gender. Sputum was lysed in TRIzol immediately after collection and total RNA was extracted using chloroform and purified and concentrated with the RNA Clean & Concentrator kit (Zymo Research, Irvine CA, USA). Total RNA was used to construct RNA-sequencing library using the Ovation Human FFPE RNA-Seq System (NuGen, San Carlos CA, USA) and sequenced with an Illumina Hi-Seq 2500 instrument. Sequence reads were quality filtered and aligned to the human reference genome (NCBI GRCh38 build) using Tophat2. Magnitude of differential gene expression and significance was calculated using DESeq2. Sequencing data are accessible from the European Nucleotide Archive with accession number PRJEB10919. Clinical samples collection was approved by the Human Research Ethics Committee of the University of Cape Town (HREC References: 031/2012 and 568/2012) and written informed consent was obtained from all participants.

### Quantification and Statistical Analysis

#### Image Analysis

Images were analysed using the image analysis software ImageJ. Dynamic association of phagosomal markers to Mtb was analysed as previously described (Schnettger and Gutierrez, 2017) with manual corrections for spacious EGFP-Rab20 positive phagosomes. Association of a specific marker to Mtb is defined as its localisation around the bacterium. The association of different markers with Mtb was measured by automated analysis of the mean relative fluorescent marker intensity in a 2-pixel wide ring around bacteria or by counting the percentage of Mtb associated with a marker. At least 250 or 100 bacteria per biological replicate were analysed during the automated analysis or manual count respectively. Phagosomal size was measured by area of phagosomes relative to area of Mtb in the same phagosome. The total relative fluorescent intensity of bacteria per cell was analysed as previously described (Lerner et al., 2016) to measure bacterial replication.

#### Statistical Analysis

Statistical analysis was performed in Prism (GraphPad Software, v6.0d, 2013). Unless otherwise stated two conditions were compared using two-tailed Student’s t tests and three or more conditions using one-way ANOVA with Tukey’s or Dunn’s multiple comparison test where appropriate. Normal distribution of the data was tested by graphical methods (histograms of the samples data). The data from the RNA Sequencing (Figure 4I) was analysed using Wald test adjusted for Benjamini-Hochberg multiple testing. The statistical tests used and the number of biological replicates is indicated in each figure legend. A minimum of 3 independent experiments was performed.

### Data and Software Availability

All data are available upon request to the lead contact author. Sequencing data are accessible from the European Nucleotide Archive with accession number PRJEB10919.

## Author Contributions

L.S. and M.G.G. designed the research. L.S., U.R., A.R., G.P., and R.P.L. performed experiments and analyzed the data. D.B.Y. and R.J.W. analyzed the data. M.V. contributed with critical reagents. M.G.G. wrote the manuscript and L.S. prepared the figures. All authors reviewed and contributed with the final version of the manuscript.

## Supplemental Information

Document S1. Figures S1–S4

Movie S1. Dynamic Association of EGFP-Rab5 and EGFP-2xFYVE with Mtb Phagosomes Occurs in the First 5 min after Internalization, Related to Figure 1

Movie S2. Dynamic Association of EGFP-Rab20 with Mtb Phagosomes, Related to Figure 1

Movie S3. Dynamic Association of LysoTracker with Mtb Phagosomes Over 24 hr, Related to Figure 3

Document S2. Article plus Supplemental Information
