# Supplementary material for: A Rab20-Dependent Membrane Trafficking Pathway Controls M. tuberculosis Replication by Regulating Phagosome Spaciousness and Integrity
Source: Cell Host Microbe. 2017 May 10;21(5):619–628.e5. doi: 10.1016/j.chom.2017.04.004 (PMC5432432; doi:10.1016/j.chom.2017.04.004)
Supplement: Document S1. Figures S1–S4 [file mmc1.pdf]

Cell Host & Microbe, Volume *21*

Supplemental Information

A Rab20-Dependent Membrane Trafficking Pathway Controls *M. tuberculosis* Replication by Regulating Phagosome Spaciousness and Integrity

Laura Schnettger, Angela Rodgers, Urska Repnik, Rachel P. Lai, Gang Pei, Martijn Verdoes, Robert J. Wilkinson, Douglas B. Young, and Maximiliano G. Gutierrez

Figure S1

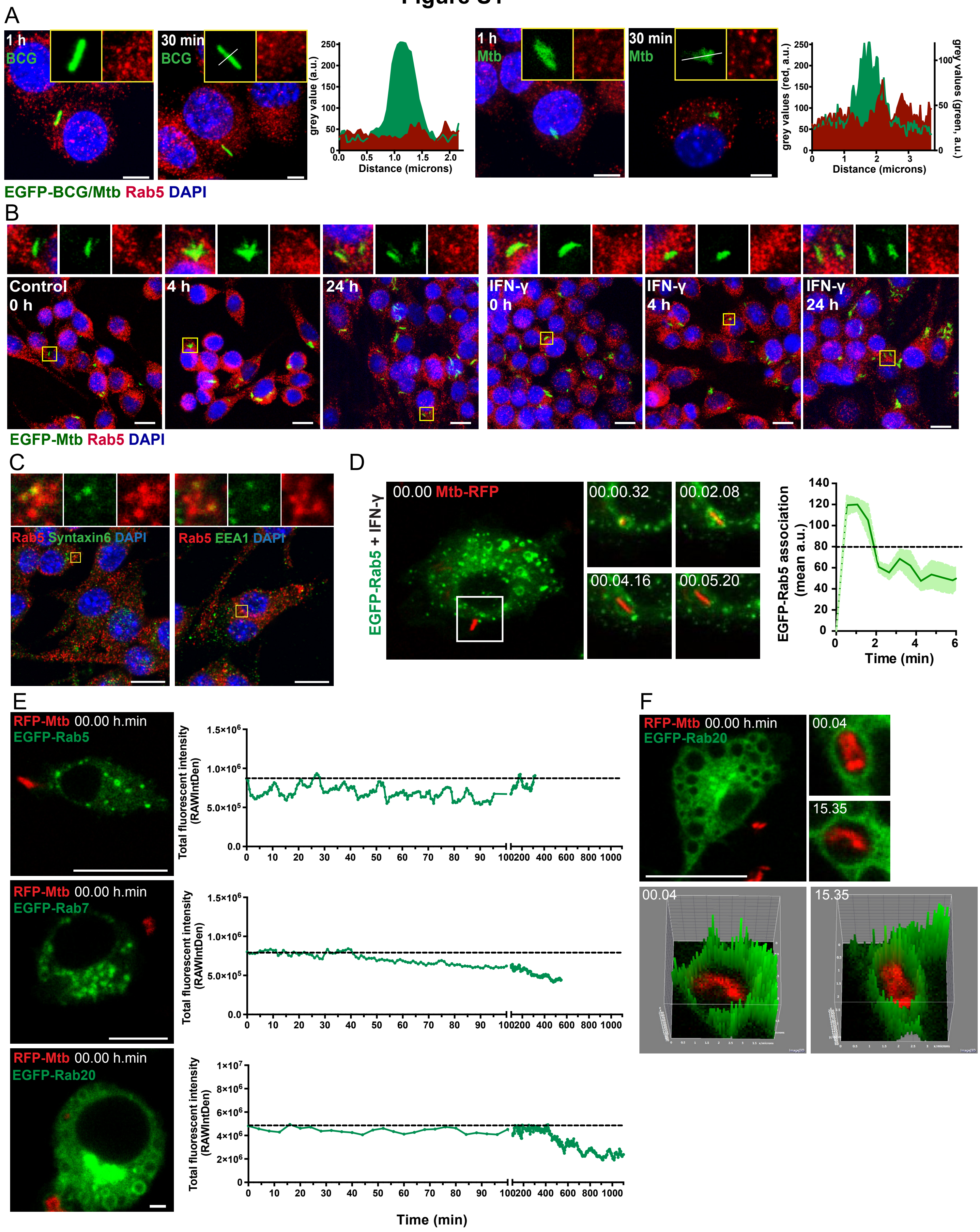
A
1 h
BCG
30 min
BCG
grey value (a.u.)
Distance (microns)
1 h
Mtb
30 min
Mtb
grey values (red, a.u.)
grey values (green, a.u.)
Distance (microns)
EGFP-BCG/Mtb Rab5 DAPI
B
Control
0 h
4 h
24 h
IFN-γ
0 h
IFN-γ
4 h
IFN-γ
24 h
EGFP-Mtb Rab5 DAPI
C
Rab5 Syntaxin6 DAPI
Rab5 EEA1 DAPI
D
EGFP-Rab5 + IFN-γ
00.00 Mtb-RFP
00.00.32
00.02.08
00.04.16
00.05.20
EGFP-Rab5 association (mean a.u.)
Time (min)
E
RFP-Mtb 00.00 h.min
EGFP-Rab5
RFP-Mtb 00.00 h.min
EGFP-Rab7
RFP-Mtb 00.00 h.min
EGFP-Rab20
Total fluorescent intensity (RAWIntDen)
Time (min)
F
RFP-Mtb 00.00 h.min
EGFP-Rab20
00.04
15.35
00.04
15.35

Figure S2

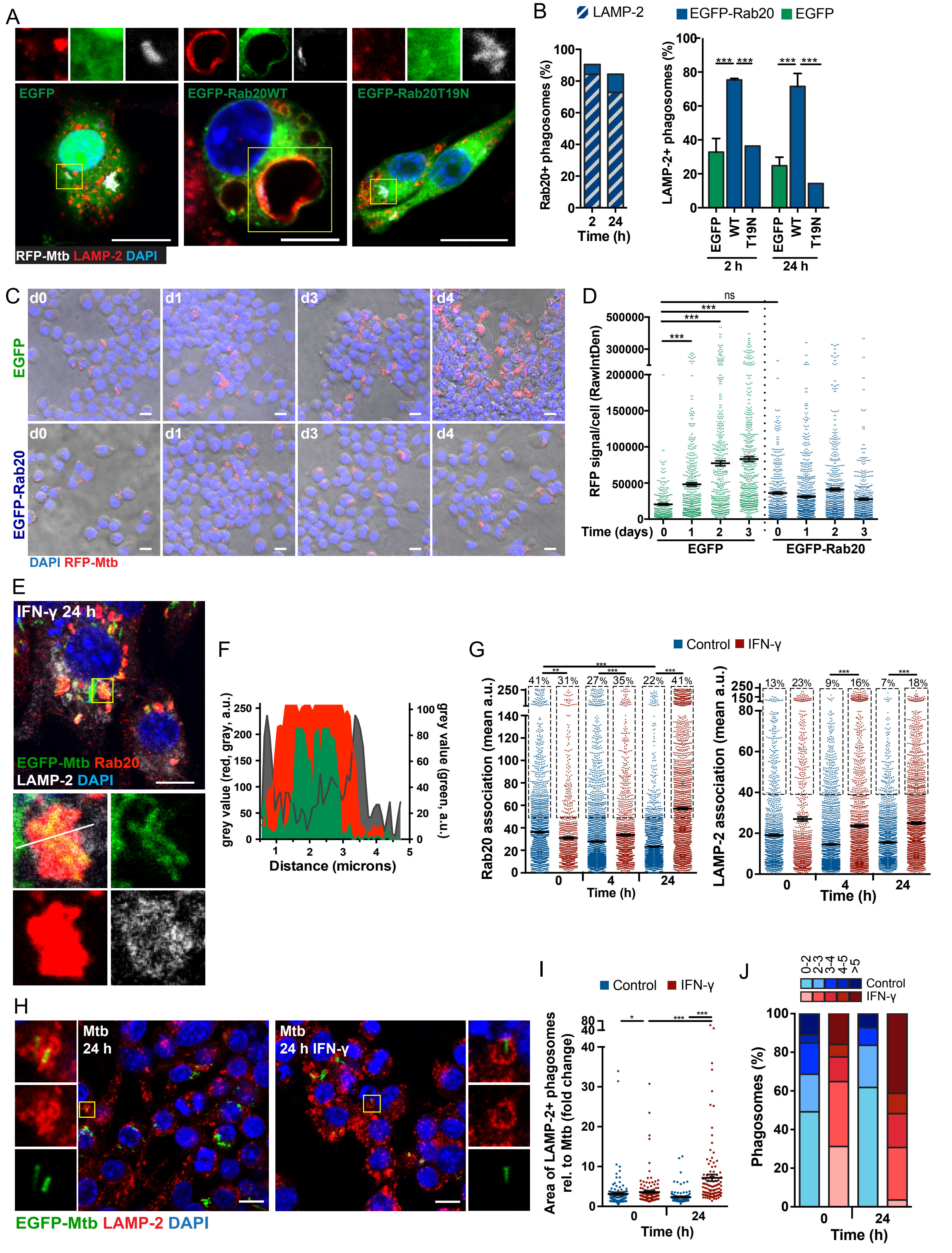
A
EGFP
EGFP-Rab20WT
EGFP-Rab20T19N
RFP-Mtb LAMP-2 DAPI
B
LAMP-2
EGFP-Rab20
EGFP
Rab20+ phagosomes (%)
LAMP-2+ phagosomes (%)
Time (h)
EGFP
WT
T19N
2 h
24 h
C
d0
d1
d3
d4
EGFP
EGFP-Rab20
DAPI RFP-Mtb
D
RFP signal/cell (RawIntDen)
Time (days)
EGFP
EGFP-Rab20
ns
E
IFN-γ 24 h
EGFP-Mtb Rab20
LAMP-2 DAPI
F
grey value (red, gray, a.u.)
grey value (green, a.u.)
Distance (microns)
G
Control
IFN-γ
Rab20 association (mean a.u.)
LAMP-2 association (mean a.u.)
Time (h)
H
Mtb
24 h
Mtb
24 h IFN-γ
EGFP-Mtb LAMP-2 DAPI
I
Control
IFN-γ
Area of LAMP-2+ phagosomes rel. to Mtb (fold change)
Time (h)
J
0-2
2-3
3-4
4-5
>5
Control
IFN-γ
Phagosomes (%)
Time (h)

Figure S3

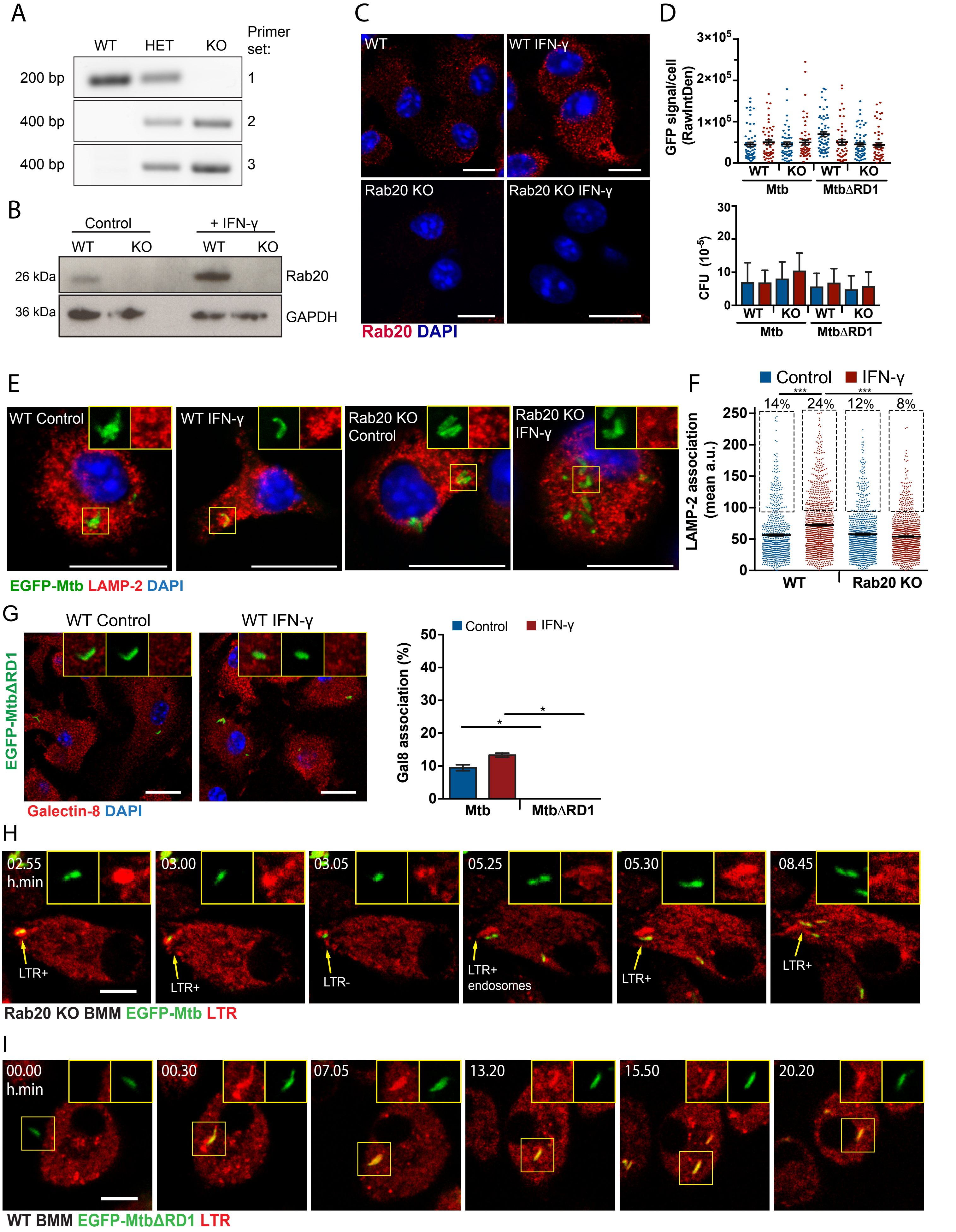
A
WT HET KO
Primer set:
200 bp 1
400 bp 2
400 bp 3
B
Control
+ IFN-γ
WT KO WT KO
26 kDa Rab20
36 kDa GAPDH
C
WT
WT IFN-γ
Rab20 KO
Rab20 KO IFN-γ
Rab20 DAPI
D
GFP signal/cell (RawIntDen)
3×10^5
2×10^5
1×10^5
0
WT KO WT KO
Mtb MtbΔRD1
CFU (10^-5)
30
20
10
0
WT KO WT KO
Mtb MtbΔRD1
E
WT Control
WT IFN-γ
Rab20 KO Control
Rab20 KO IFN-γ
EGFP-Mtb LAMP-2 DAPI
F
Control IFN-γ
***
***
14% 24% 12% 8%
LAMP-2 association (mean a.u.)
250
200
150
100
50
0
WT Rab20 KO
G
WT Control
WT IFN-γ
EGFP-MtbΔRD1
Galectin-8 DAPI
Control IFN-γ
Gal8 association (%)
50
40
30
20
10
0
*
*
Mtb MtbΔRD1
H
02.55 h.min
03.00
03.05
05.25
05.30
08.45
LTR+
LTR+
LTR-
LTR+ endosomes
LTR+
LTR+
Rab20 KO BMM EGFP-Mtb LTR
I
00.00 h.min
00.30
07.05
13.20
15.50
20.20
WT BMM EGFP-MtbΔRD1 LTR

Figure S4

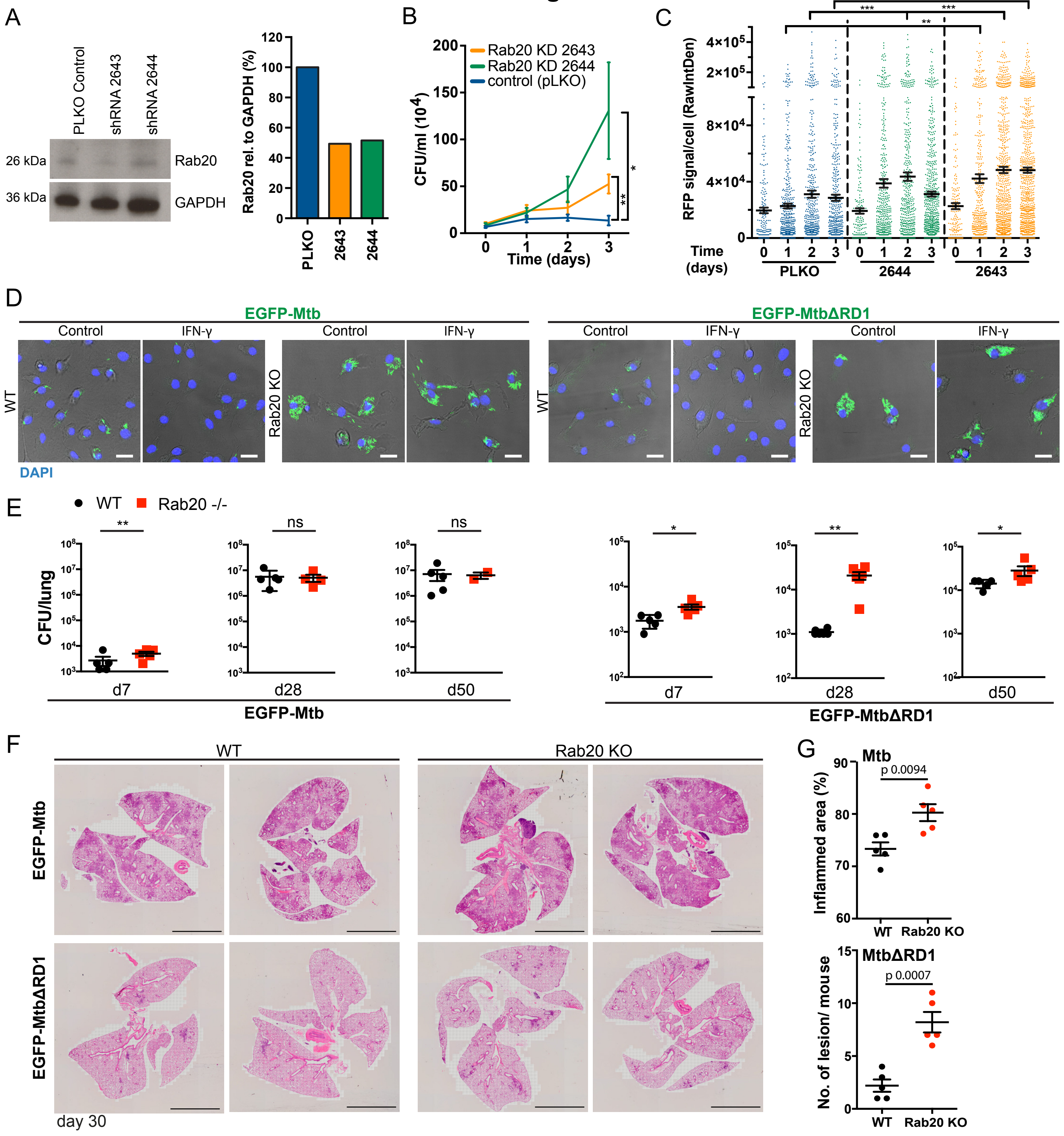
A
PLKO Control
shRNA 2643
shRNA 2644
26 kDa
36 kDa
Rab20
GAPDH
Rab20 rel. to GAPDH (%)
PLKO
2643
2644
B
Rab20 KD 2643
Rab20 KD 2644
control (pLKO)
CFU/ml (10^4)
Time (days)
C
RFP signal/cell (RawIntDen)
Time (days)
PLKO
2644
2643
D
EGFP-Mtb
EGFP-MtbΔRD1
Control
IFN-γ
WT
Rab20 KO
DAPI
E
WT
Rab20 -/-
CFU/lung
d7
d28
d50
EGFP-Mtb
EGFP-MtbΔRD1
ns
F
WT
Rab20 KO
EGFP-Mtb
EGFP-MtbΔRD1
day 30
G
Mtb
p 0.0094
Inflammed area (%)
WT
Rab20 KO
MtbΔRD1
p 0.0007
No. of lesion/ mouse

Supplemental figure legends:

Fig. S1 (related to Fig.1)- Expression of EGFP-Rab20 targets Mtb to spacious phagolysosomes and restricts Mtb replication

A- Rab5 does not associate with *M. tuberculosis* or *M. bovis* BCG after 30 min of infection. RAW264.7 macrophages were infected with EGFP-Mtb or EGFP-BCG for 30 min or 1 h. Samples were fixed and immunostained for Rab5 and the nuclear stain DAPI. Intensity profiles are shown along the white line cells infected for 30 min. Three independent experiments were performed. Scale bars: 10 μm. **B-** Endogenous Rab5 is not associated with Mtb phagosomes between 2 h of initial infection and up to 24 h after infection. Macrophages were stimulated with IFN-γ (5 ng/ml) for 24 hours prior to infection with EGFP-Mtb. Samples were immunostained for Rab5 and the nuclear stain DAPI. Three independent experiments were performed. Scale bars: 10 μm. **C-** Representative images of uninfected RAW264.7 macrophages co-immunostained for Rab5 (red) and EEA1 or syntaxin 6 (green) and DAPI. Insets show regions with colocalisation of the analysed markers. Scale bars: 10 μm. **D-** RAW264.7 macrophages were transfected with EGFP-Rab5, incubated with IFN-γ (5 ng/ml), infected with RFP-Mtb and images were taken every 32 sec for up to 10 h. Rab5 association with Mtb is shown as mean ± SEM from three independent experiments. Scale bar: 5 μm. **E-** RAW264.7 macrophages transfected with indicated expression vectors were infected with RFP-Mtb. Images were taken every 32 sec or 4 min (EGFP-Rab20) for 24 h. **A-** The total relative green fluorescent intensity was measured over the time course of the live cell imaging. Representative quantification is shown for the depicted cells. **F-** 3D Surface plots of the EGFP-Rab20 positive RFP-Mtb phagosomes at the indicated time points post-infection.

Movie S1 (related to Fig.1)- Dynamic association of EGFP-Rab5 and EGFP-2xFYVE with Mtb phagosomes occurs in the first 5 min after internalisation

RAW264.7 macrophages were transfected with EGFP-Rab5 or EGFP-2xFYVE and infected with RFP-Mtb. Images were taken every 32 sec for 24 h. Movie shows dynamic association of EGFP-Rab5 and EGFP-2xFYVE during the first 12 min after internalisation of RFP-Mtb (white arrow). Scale bars: 5 μm.

Movie S2 (related to Fig.1)- Dynamic association of EGFP-Rab20 with Mtb phagosomes

RAW264.7 macrophages were transfected with EGFP-Rab20 and infection with RFP-Mtb. Images were taken every 4 min for 24 h. Movie shows dynamic association of EGFP-Rab20 during the first 19 h after internalisation (0 min) of RFP-Mtb. Scale bar: 5 μm.

Fig. S2 (related to Fig. 1-2)- IFN-γ targets Mtb to Rab20 and LAMP-2 positive spacious phagolysosomes

A- RAW264.7 macrophages expressing EGFP, EGFP-Rab20 and EGFP-Rab20T19N infected with RFP-Mtb were fixed and stained for LAMP-2 and DAPI. Representative images are shown for 24 h of infection. Scale bars: 5 µm. **B-** Percentage of EGFP-Rab20 positive phagosomes as well as LAMP-2 positive phagosomes in both EGFP and EGFP-Rab20 expressing RAW264.7 macrophages from (A) was analysed. Quantification is shown as mean ± SEM from three independent experiments. ***$p<0.001$, one-way ANOVA with Tukey's multiple comparison test. **C-** Representative images showing growth of RFP-Mtb in RAW264.7 cells expressing EGFP or EGFP-Rab20. Samples were stained with the nuclear stain DAPI. Scale bars: 10 µm. **D-** RFP-Mtb growth from (C) was analysed by RFP signal/cell. Symbols represent individual Mtb infected cells. Quantification is shown as mean ± SEM from three independent experiments ns=statistically not significant, ***$p<0.001$, one-way ANOVA with Dunn's multiple comparison test. **E-** RAW264.7 were incubated with IFN-γ (5 ng/ml) for 24 h prior to infection with EGFP-Mtb for the indicated time points. Representative image and their corresponding 3D surface plots (image and inset, left to right) of IFN-γ stimulated RAW264.7 macrophages infected for 24 h. Cells were fixed and immunostained for Rab20, LAMP-2 and the nuclear stain DAPI. Scale bar: 10 µm. **F-** Profile of Rab20 (red) and LAMP-2 (grey) associated with EGFP-Mtb (green) along the white line in (E). **G-** Quantification of Rab20 and LAMP-2 association with EGFP-Mtb in macrophages from (E). Symbols represent single bacteria or distinct Mtb groups. Quantification is shown as mean ± SEM from three independent experiments. ***$p<0.001$, one-way ANOVA with Dunn's multiple comparison test. **H-** Representative images of LAMP-2 association with EGFP-Mtb in RAW264.7 macrophages. Scale bars: 10 µm. **I-** LAMP-2 positive phagosomal area relative to their bacterial areas from (H). Symbols represent individual phagosomes. Quantification is shown as mean ± SEM from three independent experiments. *$p<0.05$, ***$p<0.001$, one-way ANOVA with Dunn's multiple comparison test. **J-** Percentage of spacious phagosomes from (H, I). Symbols represent individual phagosomes.

Fig. S3 (related to Fig. 3)- Rab20 is required for Mtb phagosome integrity

A- Rab20 KO mice were designed as part of the European Conditional Mouse Mutagenesis Program (EUCOMM). The targeting region was design as KO first allele[3] and introduced into the C57BL/6N background. Presence of the KO targeting vector in Rab20 KO mice by PCR. Primer set 1 amplifies the WT allele, primer set 2 amplifies part of the selectable marker region of the targeting vector and primer set 3 the exon 2 of Rab20. Introduction of the targeting vector into the Rab20 gene disrupts

gene expression already in the presence of the exon 2 in the ‘knockout-first’ allele. WT – wild type, HET – heterozygous, KO – knockout. **B-** BMM from WT or Rab20 KO mice were stimulated with IFN-γ (5 ng/ml) for 24 h before cells were collected and samples prepared for Western blot analysis. GAPDH is visualised as a loading control. **C-** WT and Rab20 KO BMM were stimulated with IFN-γ (5 ng/ml) for 24 h. Samples were fixed and stained for Rab20 and the nuclear marker DAPI. Scale bars: 10 μm. **D-** Uptake of EGFP-Mtb/EGFP-MtbΔRD1 by WT and Rab20 KO BMM was measured by GFP signal/cell and CFU/ml. BMM were infected at a MOI of 0.5 for 2 hours before incubation in media for another 2 hours. Cells were then either fixed for immunofluorescence analysis or lysed and plated to measure bacterial load. Quantification is shown as mean ± SEM from three independent experiments. Symbols represent individual Mtb infected cells. **E-** WT and Rab20 KO BMM were stimulated with IFN-γ (5 ng/ml) for 24 h prior to infected with EGFP-Mtb for 24 h. BMM were fixed and immunostained for LAMP-2 and the nuclear stain DAPI. Scale bars: 5 μm. **F-** Quantification of LAMP-2 association with EGFP-Mtb from (E) is shown as mean ± SEM from three independent experiments. Symbols represent single bacteria or distinct Mtb groups. ***$p<0.001$, one-way ANOVA with Dunn’s multiple comparison test. **G-** WT and Rab20 KO BMM were stimulated with IFN-γ (5 ng/ml) for 24 h prior to infection with EGFP-Mtb or EGFP-MtbΔRD1 for 24 h. BMM were immunostained for galectin-8 and the nuclear stain DAPI. Scale bars: 10 μm. Quantification is shown as mean ± SEM from three independent experiments. *$p<0.05$, one-way ANOVA with Tukey’s multiple comparison test. **H-I-** WT or Rab20 KO BMM were incubated with 50 nM LysoTracker-Red (LTR) for 1 h before infection with EGFP-Mtb or EGFP-MtbΔRD1 and followed by live cell imaging for 24 h, with acquisition of an image every 5 min. Representative images for LTR association, loss and subsequent re-association with EGFP-Mtb in Rab20 KO BMM (H) and continuous LTR association with EGFP-MtbΔRD1 in WT BMM (I). Three independent experiments were performed. Scale bars: 5 μm.

Movie S3 (related to Fig. 3)- Dynamic association of LysoTracker with Mtb phagosomes over 24 h

WT BMM were incubated with 50 nM LysoTracker-Red (LTR) for 1 h before infection with EGFP-Mtb and followed by live cell imaging for 24 h, with acquisition of an image every 5 min. Movie shows retention, loss and subsequent re-acquisition of LTR to EGFP-Mtb phagosomes during the first 13 h after internalisation (0 min). Before LTR re-acquisition, LTR positive endosomes are observed close to bacteria. Scale bars: 5 μm.

Fig. S4 (related to Fig. 4)- Mtb replication in Rab20 KD RAW264.7 macrophages and Rab20 KO BMM

A- Western blot of RAW264.7 cells expressing scrambled shRNA (PLKO) or shRNA against Rab20 (2644/2643). Rab20 levels are shown relative to GAPDH from one experiment out of three performed. **B-** RFP-Mtb growth was analysed by CFU/ml in RAW264.7 cells expressing shRNA against Rab20 (2644/2643). Quantification is shown as mean ± SEM from three independent experiments. *p<0.05, **p<0.01, unpaired parametric Student's t test. **C-** RFP-Mtb growth was analysed by fluorescent signal/cell. Symbols represent individual Mtb infected cells. Quantification is shown as mean ± SEM from three independent experiments. **p<0.01, ***p<0.001, one-way ANOVA with Dunn's multiple comparison test. **D-** WT and Rab20 KO BMM were incubated with IFN-γ (5 ng/ml) for 24 h prior to infection with EGFP-Mtb or EGFP-MtbΔRD1 at a MOI of 0.5 for 3 days. Representative images showing growth of EGFP-Mtb/ EGFP-MtbΔRD1 in WT and Rab20 KO BMM at 3 days post infection. Scale bars: 10 µm. **E**- Growth of EGFP-Mtb/EGFP-MtbΔRD1 in lungs of WT and Rab20$^{-/-}$ mice. Data shows mean ± SEM from one representative experiment out of two. Symbols represent individual mice. *p<0.05, **p<0.01, ns=not significant, 2-tailed Student's t test. **F-** H&E stained lung sections of WT and Rab20$^{-/-}$ mice infected with EGFP-Mtb/EGFP-MtbΔRD1 for 30 days. Scale bars: 4 mm. **G-** Inflamed area or number of lesions in lungs of WT or Rab20$^{-/-}$ mice infected with EGFP-Mtb/ EGFP-MtbΔRD1 for 30 days from (F). Quantification is shown as mean ± SEM from one experiment. Symbols represent individual mice. Parametric unpaired Student's t-test.
