# Supplementary figures and images for: A Rab20-Dependent Membrane Trafficking Pathway Controls M. tuberculosis Replication by Regulating Phagosome Spaciousness and Integrity
Source: Cell Host Microbe. 2017 May 10;21(5):619–628.e5. doi: 10.1016/j.chom.2017.04.004 (PMC5432432; doi:10.1016/j.chom.2017.04.004)

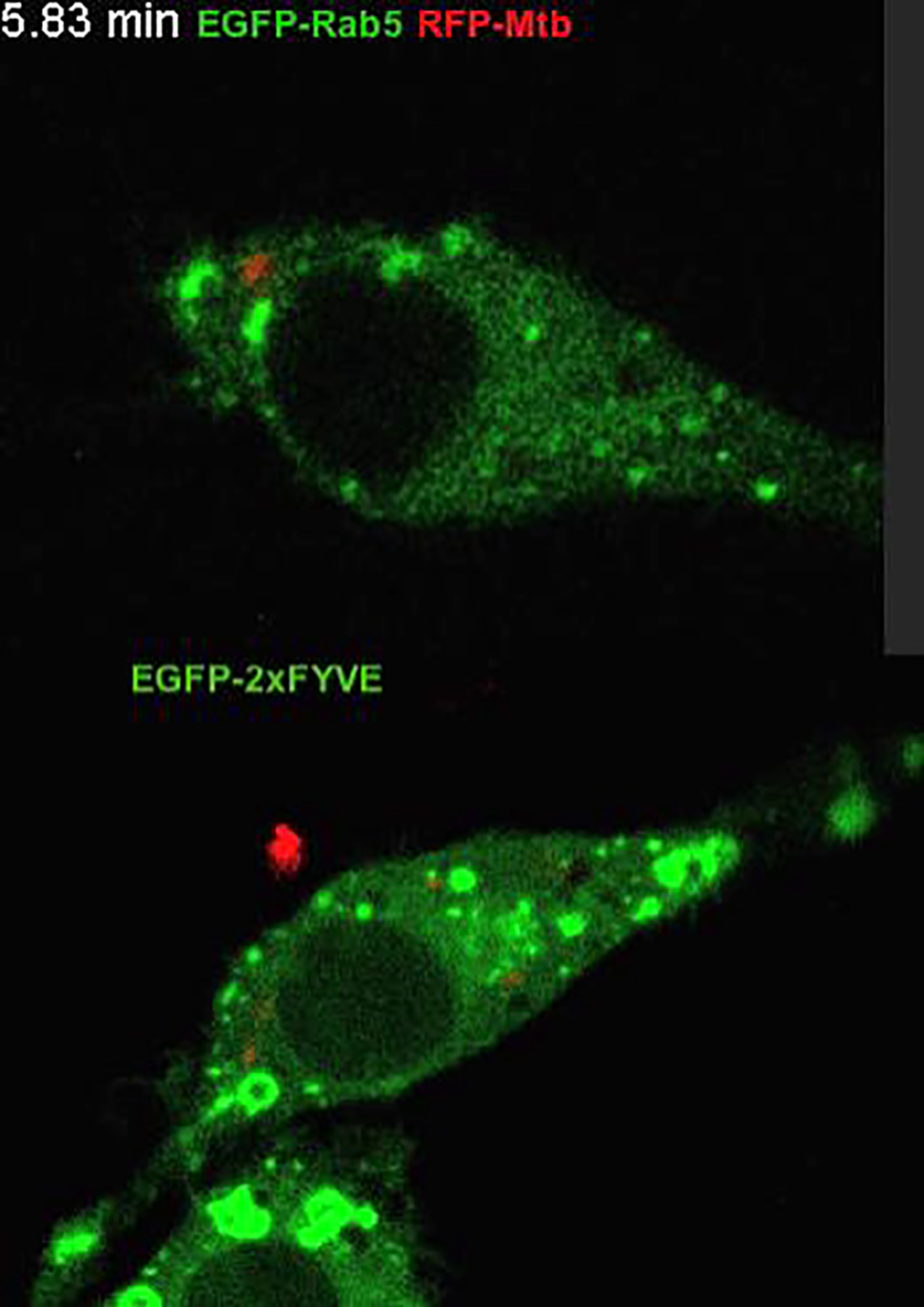

Supplement: Movie S1. Dynamic Association of EGFP-Rab5 and EGFP-2xFYVE with Mtb Phagosomes Occurs in the First 5 min after Internalization, Related to Figure 1 [file mmc2.jpg]

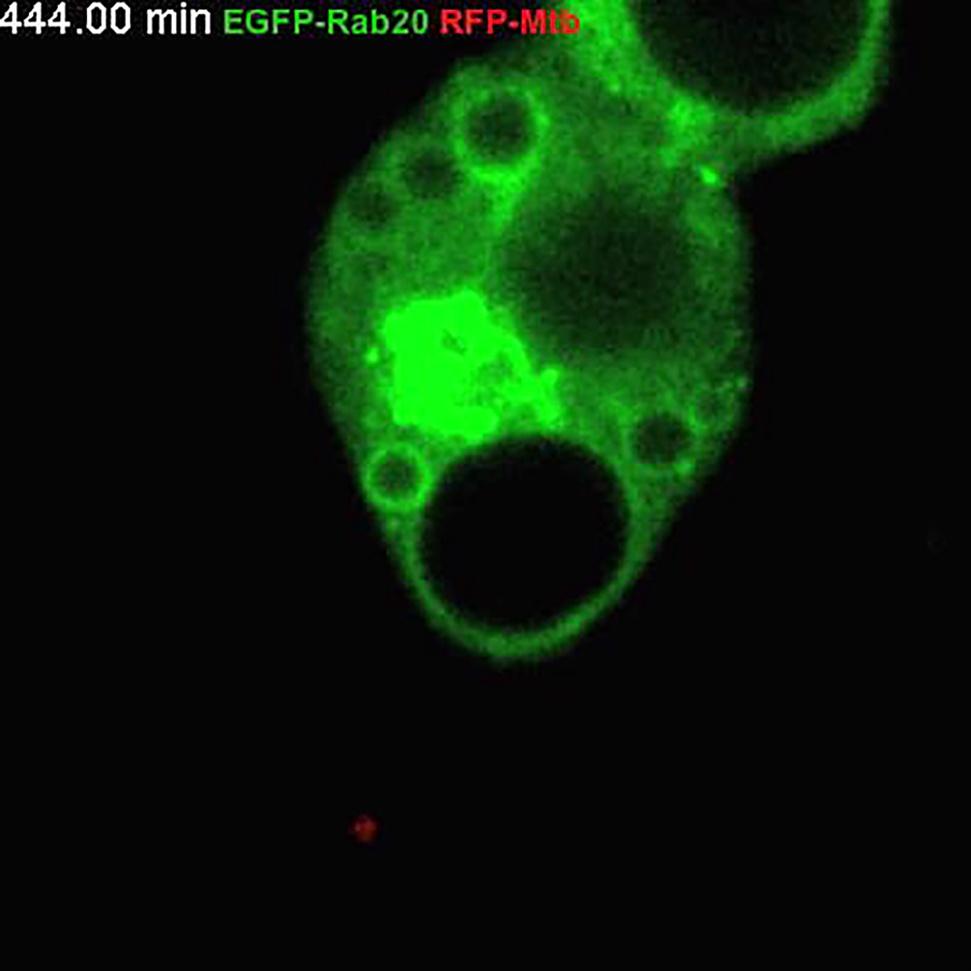

Supplement: Movie S2. Dynamic Association of EGFP-Rab20 with Mtb Phagosomes, Related to Figure 1 [file mmc3.jpg]

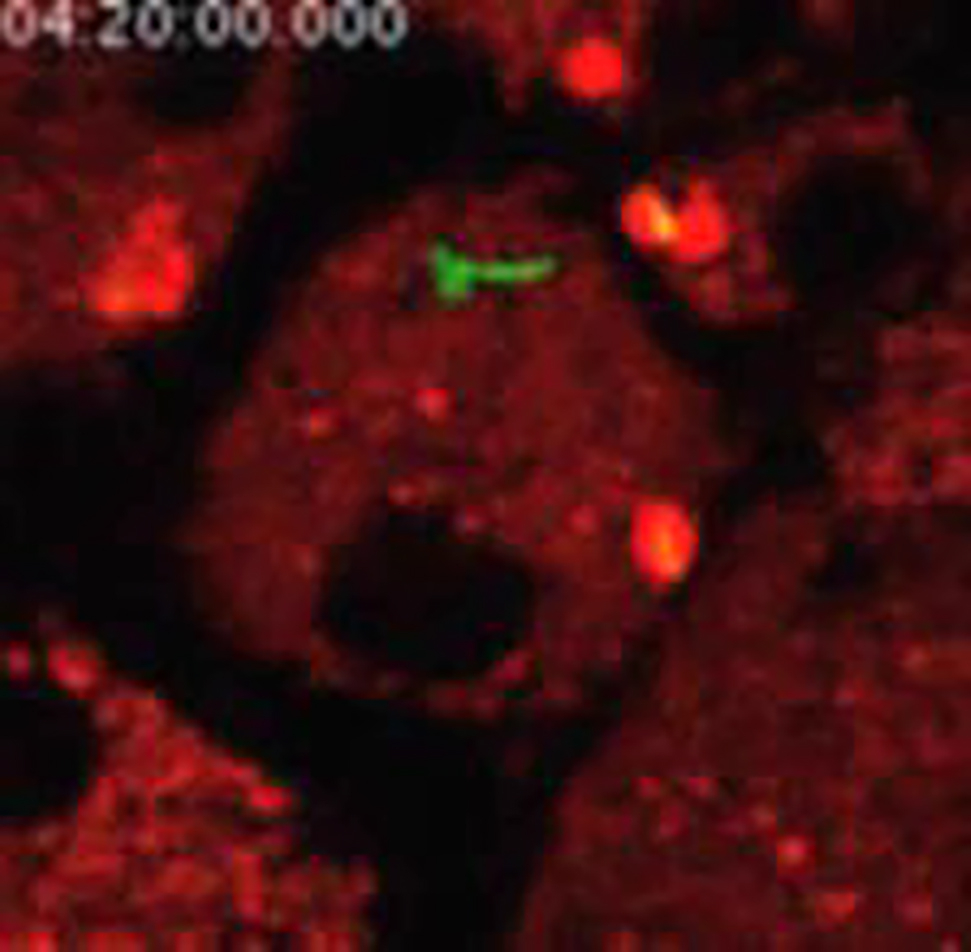

Supplement: Movie S3. Dynamic Association of LysoTracker with Mtb Phagosomes Over 24 hr, Related to Figure 3 [file mmc4.jpg]
